# Indoor hydroponic vegetable gardening to improve mental health and quality of life in cancer patients: a pilot study

**DOI:** 10.3389/fpubh.2025.1670698

**Published:** 2025-10-15

**Authors:** Taehyun Roh, Laura Ashley Verzwyvelt, Anisha Aggarwal, Raj Satkunasivam, Nishat Tasnim Hasan, Nusrat Fahmida Trisha, Charles Hall

**Affiliations:** ^1^School of Public Health, Texas A&M University, College Station, TX, United States; ^2^Houston Methodist Hospital, Houston, TX, United States; ^3^College of Agriculture and Life Sciences, Texas A&M University, College Station, TX, United States

**Keywords:** cancer patients, hydroponic gardening, mental health, quality of life, intervention

## Abstract

**Background:**

Cancer patients experience significant psychological and physiological challenges, affecting their treatment outcomes and overall wellbeing. Traditional gardening benefits mental health and quality of life but is often impractical, requiring alternatives. This pilot study evaluated the impact of indoor hydroponic gardening on cancer patients’ mental health and quality of life.

**Methods:**

A case-crossover pilot study included 36 adult cancer patients from the Houston Methodist Cancer Center, with participants serving as their own control through repeated measurements. Participants received AeroGarden hydroponic systems and engaged in an 8-week gardening intervention. Mental wellbeing, mental distress, quality of life, fruit and vegetable consumption, and pain management were assessed at baseline, 4 weeks, and 8 weeks using validated scales. Data were analyzed using Generalized Estimating Equations (GEE) to account for within-subject correlations over time.

**Results:**

The study included 36 cancer patients with a mean age of 57.5 years. Significant improvements were observed in mental wellbeing scores (*p*-trend = 0.042), depression subscale scores (*p*-trend = 0.003), and global quality of life (*p*-trend < 0.001) over the 8 weeks. Emotional and social functioning scores also improved significantly (*p*-trend = 0.001 and *p*-trend = 0.010, respectively), along with increased fruit and vegetable intake (*p*-trend = 0.028). While overall pain management scores showed a decreasing trend, these changes were not statistically significant.

**Conclusion:**

This study demonstrates that indoor hydroponic vegetable gardening can significantly improve mental health and quality of life in cancer patients, suggesting it as an alternative to traditional gardening. Future studies with larger sample sizes and longer follow-up periods are needed to confirm these findings and explore long-term benefits.

## 1 Introduction

Cancer is the second leading cause of mortality in the United States and a significant global public health challenge. In 2023, there were an estimated 1.9 million new cases of cancer, highlighting the substantial burden of managing this disease (1). Cancer management encompasses more than just medical treatment, as patients face various psychological, behavioral, and physiological challenges. Notably, the prevalence of depression among cancer patients is high, with a recent study reporting a 25% prevalence in the US (2). Depression can impede patients’ ability to make informed treatment decisions, potentially reducing acceptance of adjuvant therapies and increasing unplanned treatment interruptions, ultimately affecting their recovery (3, 4). Research indicates that cancer patients with depression face higher risks of recurrence and reduced survival rates. A meta-analysis found that depression is associated with a 24% higher risk of cancer recurrence and a 29% higher risk of cancer-specific mortality in breast cancer (5). Additionally, pain is a common and distressing symptom experienced by 66% of cancer patients, with its perception strongly influenced by emotional and cognitive factors, underscoring the connection between mental and physical health (6, 7). These challenges significantly impact overall wellbeing by compromising health-related quality of life, limiting social engagement, and delaying the resumption of work-related activities (8, 9).

To address these challenges, the concept of ‘social prescribing’ has gained traction, offering innovative and holistic approaches to enhance health and quality of life (10). Social prescribing involves the referral of patients to non-clinical services to support their physical and mental health (11). Among these approaches, gardening has been extensively shown to improve physical and mental health while enhancing overall quality of life, serving as a cost-effective and active horticultural intervention. Previous studies have demonstrated that gardening alleviates anxiety, stress, anger, fatigue, and symptoms of major depressive disorder, ultimately enhancing psychosocial wellbeing (12, 13). These benefits include reductions in body mass index, blood pressure, and reliance on pain medication in the older adults (14, 15). In healthcare settings, exposure to plants and flowers has been associated with improved patient outcomes, shorter hospitalizations, reduced pain, anxiety, and fatigue, and higher satisfaction levels (16). Specifically, vegetable gardening has emerged as a holistic approach, improving physical activity, body weight status, and psychosocial wellbeing in breast cancer survivors (17). It encourages healthier dietary choices by increasing fruit and vegetable consumption, fostering motivation, feelings of nurture, achievement, and life satisfaction in cancer survivors (18).

While regular outdoor gardening is recognized as an effective means to improve mental health through moderate physical activity and contact with nature, it is often impractical for some populations, particularly those in urban or low-socioeconomic status communities due to physical space constraints (19). This necessitates alternative gardening methods, such as hydroponics. Hydroponics, a soilless gardening method that involves placing plants in water and directly providing soluble nutrients to their roots, allows cultivation in confined spaces (20). Unlike traditional outdoor gardening, hydroponic gardening reduces risks associated with external factors such as geographical conditions, weeds, insects, and soil-borne diseases, eliminating potential exposure to toxic chemicals like herbicides and fertilizers (21). Additionally, hydroponic gardening is not constrained by climate or weather, making it suitable for engagement in any season (22).

In this study, we conducted a participatory intervention to assess the impact of indoor hydroponic vegetable gardening on the mental health and quality of life of cancer patients. This is the first participatory intervention exploring the potential benefits of indoor hydroponic gardening for this specific population.

## 2 Methods

### 2.1 Study design and population

**Figure 1:**
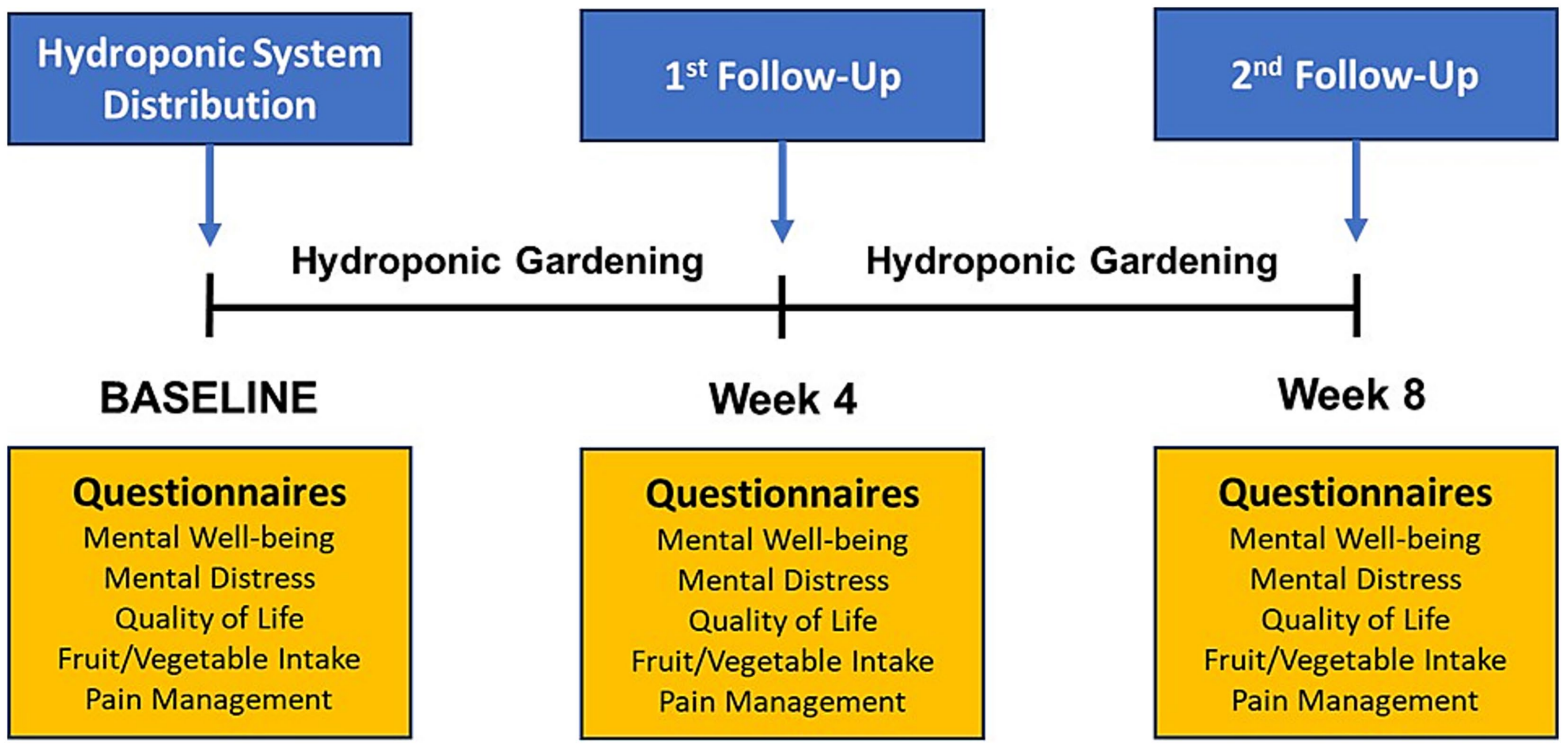
Overview of the intervention and outcome measurement timeline.

A case-crossover study evaluated the effectiveness of a hydroponic gardening intervention in improving the mental health and quality of life of cancer patients from October 2022 to September 2023. Figure 1 presents an overview of the intervention and outcome measurement timeline. Convenience sampling was used to recruit adult cancer patients from the Outpatient Infusion Center at Houston Methodist Cancer Center (HMCC). Inclusion criteria included active adult patients aged ≥ 18 years with various types and stages of cancer who had completed at least one cycle of chemotherapy and were on 14- or 28-day infusion therapy cycles to facilitate outcome measurements using surveys at 4 and 8 weeks. No specific exclusion criteria were applied since this was a feasibility study. All participants received the hydroponic gardening intervention and served as their own control for pre- and post-intervention comparisons. A total of 43 participants enrolled in the study, and 7 dropped out after the baseline survey, leaving 36 participants in the study. The 7 participants who dropped out were not systematically different from the 36 who completed the study, showing a similar distribution by sex (3 males and 4 females), race/ethnicity (5 Whites and 2 Hispanics), and mean age (60 years).

The study protocol was approved by the Institutional Review Board (IRB) of Texas A&M University (IRB ID IRB2020-1506) and the Houston Methodist Cancer Center (IRB ID PRO00031657). These approvals ensured that the study adhered to ethical standards and regulations for research involving human subjects. Written informed consent was obtained from all participants before collecting baseline data.

### 2.2 Intervention

Participants were provided with AeroGarden hydroponic vegetable gardening systems (model No. 100641) upon completing baseline surveys. This system includes a growing container, an LED grow light, liquid plant food nutrients, and 12 pod seed kits for heirloom salad greens. The AeroGarden system is designed to be user-friendly, making it accessible even for individuals with no prior gardening experience. Over an 8-week period, participants independently planted the seeds, nurtured the plants, and harvested them using the hydroponic systems at home. They were encouraged to follow a routine that involved checking the water levels, adding nutrients as required, and ensuring the LED light operated on a schedule to optimize plant growth. Manufacturer’s manuals and instructional materials were also provided to guide participants through each step of the process. These materials included detailed instructions on setting up the system, tips for successful planting and maintenance, and troubleshooting advice for common issues. Kit usage was assessed during follow-up visits through participant self-report, and all participants verbally confirmed that they had grown vegetables using the system.

### 2.3 Measures and data collection

Demographic and baseline data were collected at the start of the study, including age, sex, race/ethnicity, socioeconomic status, type of insurance, medication for psychological issues, residence type, prior engagement in gardening, and weekly outdoor activity. Mental wellbeing, mental distress, quality of life, fruit and vegetable consumption, and pain management were assessed at baseline, 4 weeks, and 8 weeks during visits to the outpatient infusion center.

Mental wellbeing was assessed using the Warwick-Edinburgh Mental Wellbeing Scale (WEMWBS), which consists of 14 positively worded items covering positive emotions, life satisfaction, self-esteem, resilience, and relationships (23). Respondents rated how often they experienced each statement in the past 2 weeks using a 5-point scale (1–5), ranging from ‘none of the time’ to ‘all of the time.’ The scale ranges from 14 to 70, with higher scores indicating better mental wellbeing.

Mental distress was measured using the Depression, Anxiety, and Stress Scale (DASS-21), which includes 21 items across three subscales: 7 items for each depression, anxiety, and stress (24). Respondents rated the frequency and severity of their experiences of each symptom over the past week using a 4-point Likert scale (0–3), from ‘not at all’ to ‘most of the time.’ Scores for 7 items in each subscale were then totaled separately to provide a comprehensive score for depression, anxiety, and stress.

Quality of life was assessed using the European Organization for Research and Treatment of Cancer Quality of Life Questionnaire (EORTC QLQ-C30), covering overall quality of life, functional domains, and common symptoms (25). The questionnaire includes five key functional domains: physical, role, cognitive, emotional, and social functioning, which assess an individual’s ability to perform physical activities, fulfill life roles, manage emotions, think clearly, and interact socially, respectively. Respondents rated their experiences on these scales and items, typically over the past week, using 4-point Likert scales (1–4) with response options such as ‘Not at all,’ ‘A little,’ ‘Quite a bit,’ and ‘Very much.’ with higher scores indicating better functioning. Scores for global quality of life were measured on a scale of 1 (very poor) to 7 (excellent).

Fruit and vegetable consumption was measured using the Behavioral Risk Factor Surveillance System (BRFSS) fruit and vegetable module, assessing intake frequency of six items: 100% fruit juice, fruit, beans, orange-colored vegetables, dark leafy vegetables, and other vegetables (26). It demonstrates moderate validity and reliability, allowing respondents to report intake frequency in terms of daily, weekly, or monthly consumption.

The Short-Form Brief Pain Inventory (SF-BPI) was used to assess the severity and impact of pain, especially in individuals with chronic pain conditions (27). It assesses pain intensity using a numerical rating scale (NRS) ranging from 0 to 10, where 0 represents ‘no pain’ and 10 represents ‘pain as bad as you can imagine.’ Additionally, it gauges the extent to which pain interferes with general activity, mood, walking ability, work, relationships with other people, sleep, and enjoyment of life on a scale from 0 (no interference) to 10 (complete interference).

### 2.4 Statistical analysis

Descriptive statistics summarized demographic and behavioral characteristics to provide a comprehensive overview of the participant population and their baseline characteristics, with means and standard deviations for continuous variables and frequencies and percentages for categorical variables. Changes in health-related outcomes (mental wellbeing, distress, quality of life, fruit and vegetable consumption, and pain management) at baseline, 4 weeks, and 8 weeks were assessed using Generalized Estimating Equations (GEE) to account for within-subject correlations over time. The GEE approach is particularly suitable for repeated measures data, as it adjusts for the correlation between repeated observations from the same subject, providing more accurate standard error estimates and confidence intervals. The GEE model was implemented using PROC GENMOD with the REPEATED statement and subject identifier for the repeated measurements of questionnaire scores from each subject, allowing the use of robust error variances to estimate confidence intervals. This method ensures that the analysis accounts for the variability within individual participants over time. Models were adjusted for potential confounders including age, sex, race/ethnicity (White and non-White), education level (high school or less, some college or above), income, prior gardening engagement, and time spent outdoors in a week. All analyses were conducted using SAS software (version 9.4; SAS Institute Inc., Cary, NC, USA), with significance set at *p* < 0.05.

## 3 Results

Among the 36 participants in the study, 47.2% were men and 52.8% were women, with an average age of 57.5 years. The majority of participants were White (58.3%) and Hispanic (30.6%). Most participants had some college education or higher. In terms of insurance, 58.3% had private insurance, while 36.1% had public insurance. The majority of participants (88.9%) had not taken psychiatric medication. Furthermore, 80.6% of participants lived in single-family houses, and 91.7% had a yard or space for gardening. Despite this, 69.4% of participants were not engaged in gardening, and 44.4% spent less than 1 h outdoors per week, while 25.0% spent more than 3 h outdoors per week (Table 1).

**Table 1:** Description of study participants (*n* = 36).

Characteristics	Frequency (%)	
Gender		
Men	17	(47.2)
Women	19	(52.8)
Age (Mean, SD)	57.5	(15.4)
Race/Ethnicity		
White	21	(58.3)
Hispanic	11	(30.6)
Black	3	(8.33)
Other	1	(2.78)
Education		
High school or less	8	(22.2)
Some college or above	28	(77.8)
Income		
Less than $50,000	14	(46.7)
$50,000–$70,000	9	(25.7)
More than $70,000	12	(34.3)
Insurance		
Private	21	(58.3)
Public	13	(36.1)
No	2	(5.56)
Psychiatric medication		
Yes	4	(11.1)
No	32	(88.9)
Residence		
Single family house	29	(80.6)
Apartment	6	(16.7)
Mobile home	1	(2.78)
Yard/space for gardening		
Yes	33	(91.7)
No	3	(8.3)
Gardening		
Yes	11	(30.6)
No	25	(69.4)
Time spent outdoors per week		
Less than 1 h	16	(44.4)
1–3 h	11	(30.6)
More than 3 h	9	(25.0)
Type of cancer		
Colorectal	7	(19.4)
Pancreas	7	(19.4)
Lung	5	(13.9)
Breast	3	(8.3)
Blood	2	(5.6)
Brain	2	(5.6)
Ovarian	2	(5.6)
Stomach	2	(5.6)
Other^a^	6	(16.7)
Stage of cancer		
2	4	(11.1)
3	5	(13.9)
4	20	(55.6)
Not reported	7	(19.4)

a Other includes one case each of bladder, duodenum, leg, liver, lymphoma, and myeloma.

**Figure 2:**
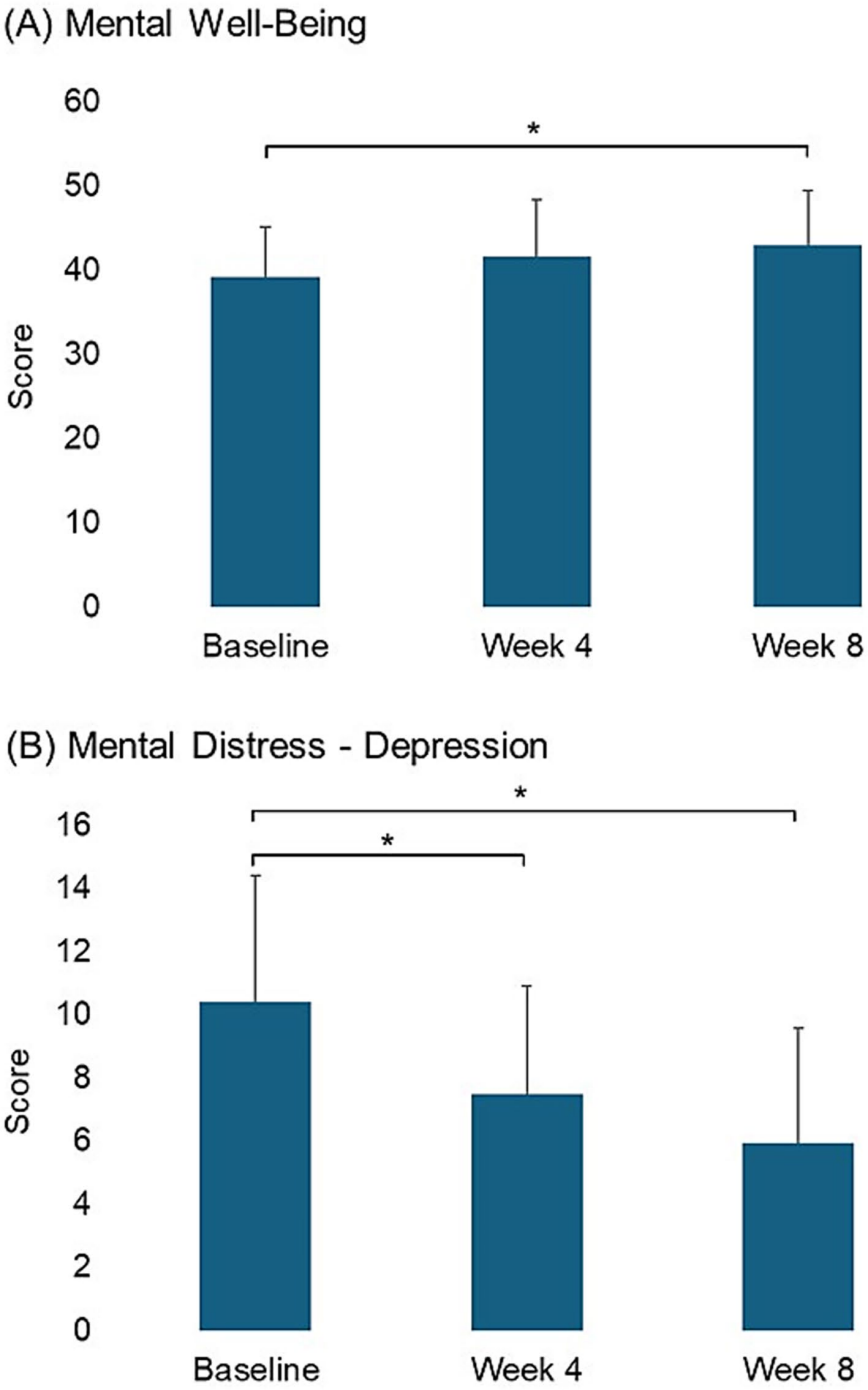
Pairwise comparison of scores in mental wellbeing and mental distress scales between time points for measures showing significant linear changes (*p < 0.05).

The scores for mental wellbeing improved steadily over the 8-week period, showing a linear improvement over the study period (*p*-trend = 0.042) (Table 2 and Figure 2). While scores on the stress and anxiety subscales did not show statistically significant changes, the depression subscale demonstrated a significant decrease over time (*p*-trend = 0.003) (Table 2), with statistically significant differences between baseline and both follow-up points (Figure 2).

**Table 2:** Adjusted^a^ least square means for mental wellbeing and distress scores over 8 weeks.

Measures	Baseline Mean (95% CI)	Week 4 Mean (95% CI)	Week 8 Mean (95% CI)	*p*-trend
Mental wellbeing	39.1 (33.1, 45.1)	41.6 (34.8, 48.4)	42.9 (36.5, 49.4)	0.042*
Mental distress				
Stress	9.65 (6.18, 13.1)	9.35 (5.47, 13.2)	8.38 (4.37, 12.4)	0.460
Depression	10.4 (6.40, 14.4)	7.51 (4.07, 10.9)	5.94 (2.32, 9.57)	0.003*
Anxiety	7.46 (5.20, 9.73)	7.56 (5.92, 9.20)	6.67 (4.62, 8.72)	0.566

a Adjusted for sex, age, race/ethnicity, education, household income, regular gardening engagement, and weekly time spent outdoors; *p < 0.05 indicates a significant linear trend over 8 weeks.

**Figure 3:**
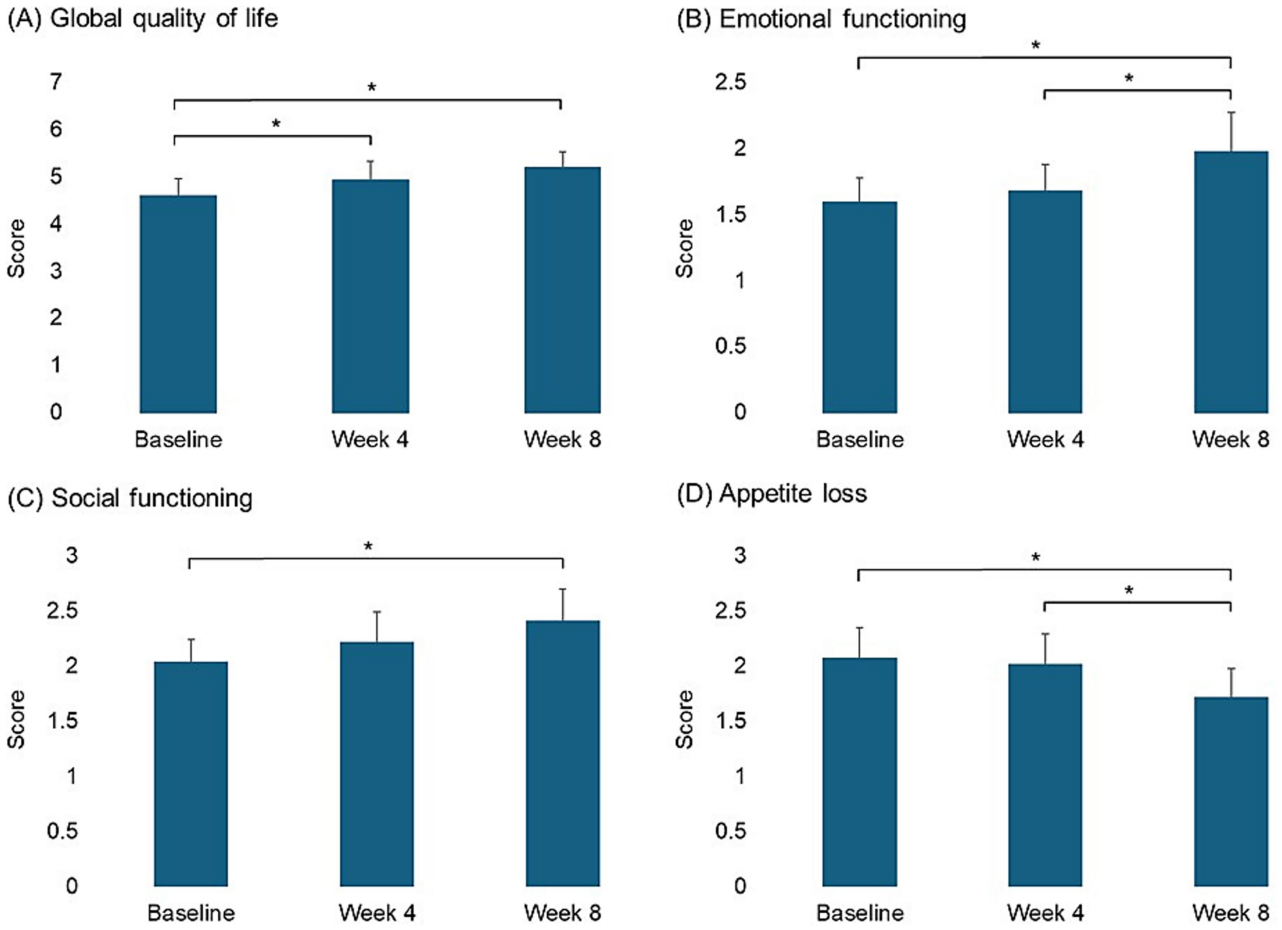
Pairwise comparison of scores in quality-of-life scales between time points for measures showing significant linear changes (*p < 0.05).

The global quality of life showed a significant improvement at week 4 (Figure 3) with an increase over the study period (*p*-trend < 0.001) (Table 3). Specifically, emotional and social functioning scores showed significant improvements at week 8 with a linear increase (*p*-trend = 0.001 and *p*-trend = 0.010, respectively), while appetite loss scores decreased significantly at week 8 with a continuous reduction in scores over the 8 weeks (*p*-trend = 0.007) (Table 3 and Figure 3).

**Table 3:** Adjusted^a^ least square means for quality-of-life scores over 8 weeks.

Measures	Baseline Mean (95% CI)	Week 4 Mean (95% CI)	Week 8 Mean (95% CI)	*p*-trend
Global quality of life	4.61 (4.25, 4.96)	4.95 (4.59, 5.32)	5.21 (4.90, 5.52)	<0.001*
Functional				
Physical	1.83 (1.63, 2.03)	1.85 (1.65, 2.04)	1.84 (1.64, 2.04)	0.932
Role	1.91 (1.69, 2.13)	2.05 (1.79, 2.32)	2.15 (1.82, 2.47)	0.141
Emotional	1.60 (1.43, 1.78)	1.68 (1.47, 1.88)	1.98 (1.69, 2.27)	0.001*
Cognitive	1.69 (1.51, 1.87)	1.69 (1.50, 1.87)	1.74 (1.52, 1.97)	0.647
Social	2.04 (1.83, 2.24)	2.22 (1.95, 2.49)	2.41 (2.12, 2.70)	0.010*
Symptom				
Fatigue	2.48 (2.20, 2.75)	2.49 (2.21, 2.76)	2.30 (2.03, 2.58)	0.075
Nausea and vomiting	1.61 (1.39, 1.83)	1.60 (1.39, 1.80)	1.53 (1.31, 1.75)	0.572
Pain	2.11 (1.78, 2.44)	2.05 (1.70, 2.40)	1.86 (1.55, 2.17)	0.109
Dyspnea	1.48 (1.27, 1.70)	1.63 (1.44, 1.82)	1.57 (1.37, 1.78)	0.533
Insomnia	2.12 (1.83, 2.41)	2.03 (1.73, 2.33)	2.01 (1.70, 2.32)	0.533
Appetite loss	2.08 (1.82, 2.35)	2.02 (1.76, 2.29)	1.72 (1.46, 1.98)	0.007*
Constipation	2.02 (1.73, 2.31)	1.96 (1.66, 2.27)	1.84 (1.59, 2.09)	0.250
Diarrhea	1.74 (1.44, 2.04)	1.71 (1.42, 2.00)	1.70 (1.37, 2.03)	0.829

a Adjusted for sex, age, race/ethnicity, education, household income, regular gardening engagement, and weekly time spent outdoors; *p < 0.05 indicates a significant linear trend over 8 weeks.

The five functional domains—physical, role, cognitive, emotional, and social—reflect the ability to perform physical activities, fulfill life roles, manage emotions, think clearly, and interact socially, respectively.

**Figure 4:**
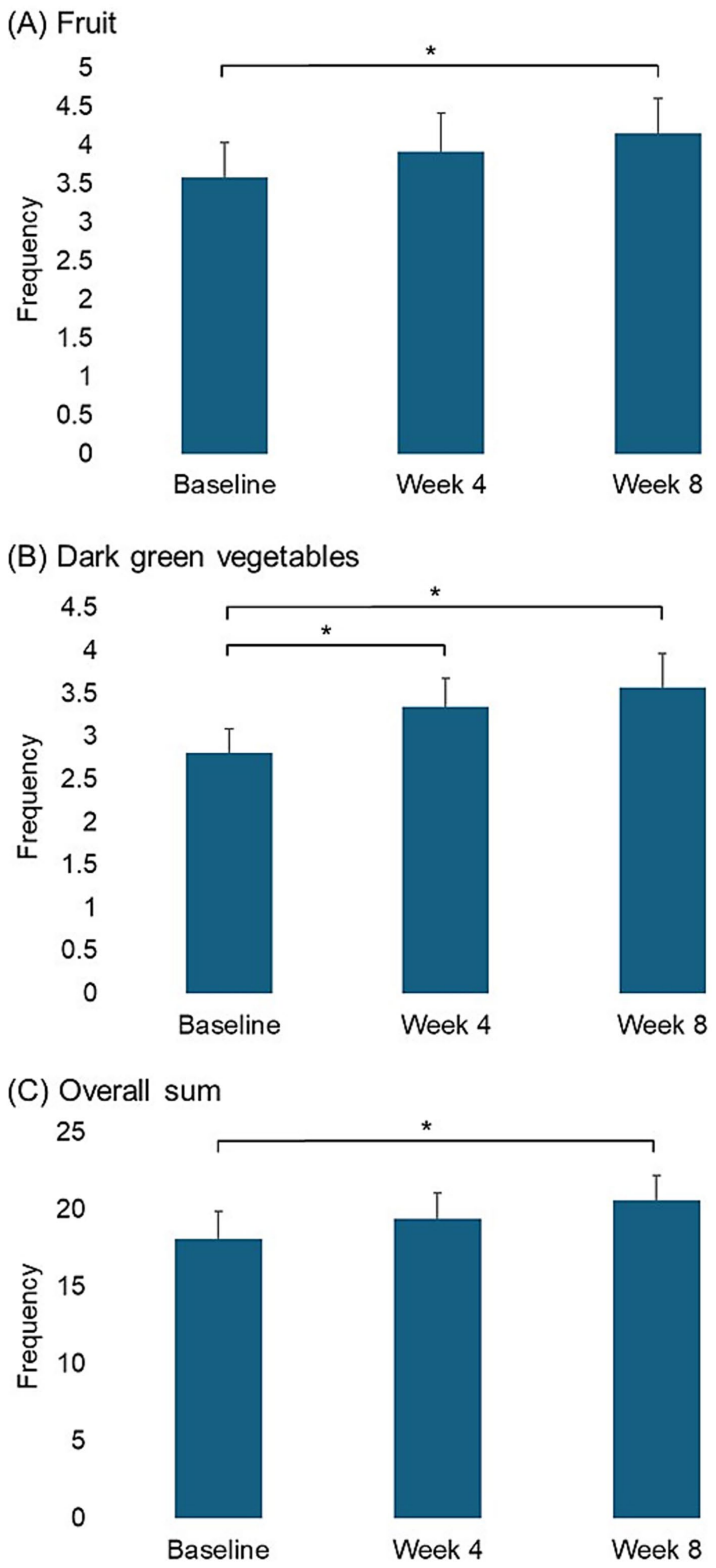
Pairwise comparison of frequencies of fruit and vegetable intake between time points for measures showing significant linear changes (*p < 0.05).

Fruit and vegetable intake increased over time (*p*-trend = 0.028) (Table 4), with a significant improvement at week 8 (Figure 4). Notably, the intake of fruits and dark green leafy vegetables significantly increased at week 8 and week 4, respectively, over the study period (*p*-trend = 0.027 and *p*-trend < 0.001, respectively) (Table 4 and Figure 4). Scores for pain management showed decreasing trends, but these changes were not statistically significant (Table 5).

**Table 4:** Adjusted^a^ least square means for frequency of fruit and vegetable intake over 8 weeks.

Measures	Baseline Mean (95% CI)	Week 4 Mean (95% CI)	Week 8 Mean (95% CI)	*p*-trend
Fruit juice	3.04 (2.50, 3.58)	3.01 (2.40, 3.63)	3.50 (2.93, 4.07)	0.229
Fruit	3.58 (3.12, 4.03)	3.92 (3.42, 4.42)	4.15 (3.69, 4.61)	0.027*
Legume	3.06 (2.53, 3.59)	2.97 (2.58, 3.36)	3.17 (2.70, 3.63)	0.750
Dark green vegetables	2.81 (2.53, 3.09)	3.35 (3.03, 3.68)	3.57 (3.17, 3.97)	<0.001*
Orange-colored vegetables	2.46 (2.10, 2.83)	2.72 (2.38, 3.06)	2.71 (2.31, 3.11)	0.361
Other vegetables	3.14 (2.77, 3.51)	3.50 (3.14, 3.86)	3.71 (3.24, 4.16)	0.076
Overall	18.1 (16.3, 19.9)	19.4 (17.7, 21.1)	20.6 (19.0, 22.2)	0.028*

a Adjusted for sex, age, race/ethnicity, education, household income, regular gardening engagement, and weekly time spent outdoors; *p < 0.05 indicates a significant linear trend over 8 weeks.

**Table 5:** Adjusted^a^ least square means for pain management scores over 8 weeks.

Measures	Baseline Mean (95% CI)	Week 4 Mean (95% CI)	Week 8 Mean (95% CI)	*p*-trend
Worst pain	4.46 (3.43, 5.49)	4.39 (3.39, 5.40)	3.92 (2.80, 5.05)	0.378
Least pain	2.52 (1.83, 3.21)	2.59 (1.91, 3.26)	2.75 (1.91, 3.58)	0.618
Pain on average	3.61 (2.83, 4.38)	3.39 (2.74, 4.05)	2.90 (2.05, 3.75)	0.122
Activity interfered due to pain	4.27 (3.39, 5.16)	3.69 (2.85, 4.53)	3.84 (2.83, 4.84)	0.297

a Adjusted for sex, age, race/ethnicity, education, household income, regular gardening engagement, and weekly time spent outdoors; *p < 0.05 indicates a significant linear trend over 8 weeks.

## 4 Discussion

The present study aimed to investigate the impact of indoor hydroponic vegetable gardening on the mental health and quality of life of cancer patients. The findings highlight the potential benefits of this intervention, particularly in improving mental wellbeing and reducing symptoms of depression. Especially, mental wellbeing scores increased by 3.8 points, exceeding the 3-point threshold considered clinically meaningful (28). In addition, significant improvements were observed in emotional and social functioning scores, along with a decrease in appetite loss scores and an increase in fruit and vegetable intake, especially in the consumption of fruits and dark green leafy vegetables. These findings suggest that indoor hydroponic gardening not only positively influences mental wellbeing but also enhances the overall quality of life of cancer patients.

Previous studies have further demonstrated the benefits of gardening on the mental health and quality of life of patients. For instance, a study found that cancer patients who engaged in gardening activities experienced significant improvements in mood and reductions in stress, anxiety, and depression (29). Similarly, another study reported that horticultural therapy, including gardening, led to significant improvements in anxiety, depression, mood disturbances, and perceived stress among cancer patients (2, 3). A more recent study found that gardening therapy significantly improved vitality, lower body flexibility, agility, and dynamic balance, contributing to a better overall quality of life for cancer patients (30). It has been proposed that gardening could modulate the autonomic nervous system by enhancing parasympathetic activity and suppressing sympathetic activity, leading to reduced stress and increased feelings of tranquility (31, 32). Beyond these physiological pathways, psychological mechanisms may also underlie the benefits of therapeutic horticulture. The nurturing of plants fosters a sense of responsibility, control, and achievement, which is particularly valuable for patients who may feel a loss of autonomy during treatment (33, 34). Gardening also provides multisensory engagement, including sight, touch, and smell, which supports mindfulness, cognitive restoration, and stress relief, consistent with attention restoration and biophilia theories (35, 36).

Our study’s findings align with previous research showing that participating in gardening activities significantly enhances dietary consumption patterns. For example, a study found that gardening increases fruit and vegetable intake and promotes healthier dietary behaviors, such as adopting a plant-based diet, reducing red meat and processed food consumption, and snacking more on vegetables in cancer survivors (37, 38). Another study found that engaging in gardening activities led to higher consumption of fruits and vegetables, improved nutritional knowledge, and better overall dietary habits among participants (39, 40). Moreover, research has shown that gardening can lead to sustained dietary changes, as individuals who grow their own food are more likely to consume fresh produce regularly (41). Gardening also reinforces self-efficacy, as patients experience a direct connection between their efforts and tangible rewards, which may help sustain long-term dietary changes and healthier lifestyles (42, 43). These findings support our results, suggesting that hydroponic gardening can have a lasting positive impact on dietary behaviors and nutritional intake.

Additionally, scientific evidence supports further benefits of gardening for cancer patients and survivors, such as improved pain management, better treatment outcomes, and reduced recurrence of health issues. For instance, a study found that cancer patients who engaged in gardening reported significantly lower pain levels and used fewer pain medications compared to those who did not garden (41, 44). Another research study demonstrated that therapeutic horticulture improved overall treatment outcomes in cancer patients, including increased adherence to treatment protocols and enhanced recovery rates (45). Furthermore, engaging in regular gardening activities has been associated with a reduced risk of disease recurrence, as it promotes a healthier lifestyle through increased physical activity and better dietary habits (46). These findings highlight the comprehensive benefits of gardening, suggesting that it can play a significant role in holistic patient care and long-term health management.

These studies collectively suggest that the therapeutic effects of gardening are well-documented, and our findings extend this knowledge by highlighting the potential benefits of hydroponic gardening, which offers a viable alternative for those with limited access to traditional gardening activities. While traditional outdoor gardening is widely recognized for its benefits to mental health through moderate physical activity and interaction with nature, it can be challenging for certain groups, especially those in urban or economically disadvantaged communities, due to limited space (19). Alternatives such as community gardens and healing gardens have been suggested (47, 48); however, these options are often unavailable or limited in many communities. Additionally, they may not be as accessible and are susceptible to external challenges such as seasonal changes, climate, geographical conditions, weeds, and insects (21). Furthermore, access and engagement could be restricted by factors like the COVID-19 pandemic and the necessity of social distancing. In contrast, hydroponic gardening provides opportunities to overcome these obstacles. It also accommodates individuals of all ages, including children, the older adults, and those with disabilities (49), thereby contributing to reducing health disparities within diverse populations in any season.

This study stands out for its innovative approach, utilizing hydroponic gardening as a departure from traditional gardening studies, thereby expanding our knowledge of horticultural therapy in healthcare. The participatory intervention design, involving patients in planting and nurturing their gardens, aligns with patient-centered care principles, promoting a sense of control and empowerment, leading to better mental health outcomes (50). Moreover, the study’s use of diverse quantitative surveys offers a comprehensive understanding of hydroponic gardening’s impact on cancer patients, facilitating an exploration of various mental health, quality of life, and dietary outcomes.

However, this study has several limitations. First, the sample size was small, with only 36 cancer patients. As a result, not all covariates could be included due to the small number of subjects in each category, potentially limiting the statistical power and generalizability of the findings. Second, the 8-week intervention period may not adequately capture long-term effects, necessitating longer follow-up studies. Third, the absence of a control group in the single-arm design raises concerns about establishing causality and introduces self-selection bias, as participants may have had pre-existing interest or positive attitudes toward gardening. Fourth, kit usage was assessed solely through self-report; future studies should incorporate objective measures such as photos, activity logs, or usage tracking to better evaluate engagement. Finally, recruiting participants from a single center may restrict the applicability of the results to other cancer care settings or diverse demographic populations.

## 5 Conclusion

This study suggests that indoor hydroponic gardening can improve mental health, enhance quality of life, and positively influence eating behaviors in cancer patients, highlighting its potential as a valuable intervention in cancer care. Health professionals have an opportunity to introduce hydroponic gardening as an integral part of cancer treatment regimens, promoting mental wellbeing and overall quality of life sustainably. Furthermore, these findings can extend beyond cancer care to general populations who may lack access to conventional gardening, enabling them to increase their contact with nature and promote wellness. To build on these findings, future studies should include larger samples with control groups, account for cancer type and stage, and incorporate longer follow-up and objective engagement measures, ideally with larger randomized controlled trials.

## Data Availability

The raw data supporting the conclusions of this article will be made available by the authors, without undue reservation.
